# Glutaric Aciduria Type II With Ketosis in a Male Infant

**DOI:** 10.7759/cureus.14407

**Published:** 2021-04-10

**Authors:** Krutika Tandon, Rahul Tandon, Meet Patel, Charmy Parikh, Henil Upadhyay

**Affiliations:** 1 Pediatrics, Pramukhswami Medical College, Anand, IND

**Keywords:** glutaric aciduria type ii, organic aciduria, tandom mass spectrometry

## Abstract

Glutaric aciduria type II (GA II) also known as multiple acyl-CoA dehydrogenase deficiency is an inborn metabolic disorder belonging to the family of organic acidurias. It is a disorder that interferes with the body's ability to break down proteins and fats to produce energy. Tandem mass spectrometry (TMS) acts as a screening tool, while the diagnosis of GA-II with ketosis is confirmed by a combination of tests like organic acids, quantitative random urine, and a full urine panel. Early diagnosis, compliance to specialized diet, affordability, and regular follow-ups are required to tackle this potentially life-threatening condition. Herein, we report a case of glutaric aciduria type-II with ketosis in a 4.5 months old male infant who was managed with a low-protein diet, which was free of tryptophan, lysine, and other specific dietary supplements.

## Introduction

Glutaric aciduria type II (GA II) is an autosomal recessive disorder of fatty acid, amino-acid, and choline metabolism. It results from the deficiency of any one of the three molecules: the alpha (ETFA) or beta (ETFB) sub-units of electron transfer flavoprotein, or electron transfer flavoprotein dehydrogenase (ETFDH) [1]. The former two are associated with neonatal forms and the latter one with older age groups [2]. The three main clinical phenotypes of GA-II are the neonatal form with congenital anomalies, neonatal form without congenital anomalies, and older onset age-group with myopathic phenotype, and rarely, metabolic acidosis [2]. The clinical presentation and onset of disease may vary depending upon the location and nature of the mutations [3]. Patients of GA-II present with varying symptoms like abnormal posturing, excessive crying and irritability, and clinical signs like hypotonia, tachycardia, tachypnea, movement disorders, hypoglycemia, and often neonatal death [1,4].

## Case presentation

A 4.5-month-old male infant presented to the pediatric outpatient department (OPD) with complaints of intermittent abnormal posturing, lethargy, excessive crying for nine days, and cough with coryza for seven days. He was admitted for five days in a private hospital where-in blood investigations were done and supportive treatment in form of IV fluids and anti-convulsants were started, but as his symptoms persisted he was referred to our hospital. There were no complaints of fever, vomiting, seizures, rash, or diarrhea. The child was a firstborn out of a second-degree consanguineous marriage. There was no significant birth history, past medical history, or family history. He was fully immunized as per his age and a bacillus Calmette-Guérin (BCG) scar was present. As per the mother, developmental history was normal but due to intermittent posturing, we were not able to elicit the milestones. His vitals on admission were normal. The examination did not show any dysmorphic features. Anterior fontanelle was normal. Anthropometry was normal except for weight (less than the third centile). His central nervous system (CNS) examination showed intermittent right hemidystonic posturing and orofacial grimacing which disappeared on sleep, confirming that it was an extrapyramidal symptom. Other systemic examination findings were normal. Laboratory investigations were normal except for low Hb (8.9 gm/dL). The peripheral blood picture showed a normocytic and normochromic blood picture. Chest X-ray showed right-sided consolidation. MRI scan of the brain showed frontotemporal atrophy, dilated Sylvian fissures with open opercula typically seen as a batwing appearance. These features were suggestive of glutaric aciduria Type II. Tandem mass spectrometry, a metabolic screening tool, showed low free carnitine, acetylcarnitine, and octadecanoyl carnitine (Table 1). As it was inconclusive, we sent urine organic acids, quantitative and random urine full panel which supported the diagnosis of GA II with ketosis (Table 2). 

**Table 1:** Urine organic acids panel

Organic Acids	Result	Ref Range in %	Elevation Factor
Pyruvic Acid	57.23	4.5	12.72
Glutaric Acid	88.79	1.9	46.73
Adipic Acid	82.78	3	27.59
Suberic Acid	43.67	2.4	18.20
Sebacic Acid	54.18	2.2	24.63

**Table 2:** Blood amino acid levels

Amino Acids	Results	Units	Ref Interval
Free Carnitine (C0)	4.88	µmol/L	8-100
Acetylcarnitine (C2)	2.78	µmol/L	8-150
Octadecanoylcarnitine (C18)	0.25	µmol/L	0.6-3.50

The infant was prescribed a low protein diet free of tryptophan and lysine. Biotin, carnitine, and riboflavin were also supplemented with amino-acid modified infant formula along with other supportive treatments in form of anti-convulsants and drugs to decrease abnormal movement. At the time of discharge, the patient’s relatives were explained about his condition and poor prognosis but eventually, we lost follow-up.

## Discussion

The prevalence of GA-I is 1-in-5600 while that of GA-II is 1-in-2,00,000 in both males and females [1,2]. Patients with GA-II may have present with varied clinical manifestations depending upon their age. In the neonatal form with a complete enzymatic deficiency, the disease appears 16-48 hours after birth and shows rapid neurological deterioration. It is characterized by hypotonia, central apnea, convulsions, and neonatal death [5]. These features are also present in carnitine palmitoyltransferase deficiency-II where the main pathology is a defect in the long-chain fatty acid oxidation. In Zellweger syndrome, a rare lethal congenital syndrome, the newborn may present with features similar to GA-II. It is a condition with reduced or absent peroxisomes in the cells. Patients with GA-II who present in adolescence or adulthood with acute Reye’s syndrome-like illness with ketoacidosis and lipid storage myopathy triggered by infections or fasting have shown better outcomes [6,7].

Our patient presented with movement disorder in form of right hemidystonic posturing with orofacial grimacing, which disappeared during sleep. MRI scan of the brain showed frontotemporal atrophy, dilated Sylvian fissures with open opercula typically seen as a batwing appearance. These features were suggestive of glutaric aciduria type II. Similar features are seen in other conditions such as idiopathic external hydrocephalus, severe developmental delay, and non-accidental injury [8]. Acylcarnitine analysis in tandem mass spectrometry is the recommended diagnostic tool for GA-II. We made a diagnosis by sending urine organic acid as the tandem mass spectrum was not confirmatory. Treatment of GA-II includes a protein- and fat-restricted diet with carbohydrate, riboflavin, glycine, coenzyme Q10, and L-carnitine supplements. Riboflavin which is the precursor of flavin adenine dinucleotide (FAD) and flavin mononucleotide (FMN), is the main therapeutic agent due to its favorable effect on flavin-dependent mitochondrial enzymes [9]. A racemic mixture of sodium D, L-3-hydroxybutyrate (NaHB) is a promising therapeutic alternative as it substitutes the deficient endogenous ketone body production which is needed for energy production, and for tissue components like myelin in the central nervous system [10,11]. It is also an additional therapeutic choice for cardiomyopathy and lacks any notable adverse side effects [12,13]. As our patient belonged to low social-economic status and due to the unavailability of above mentioned therapeutic dietary options, we continued the same treatment. Another limitation noticed was the inability to perform genetic mutation tests due to financial constraints. At the time of discharge, some improvement in symptoms was observed but complete recovery was not present and we lost follow-up.

## Conclusions

This case report briefly highlights the various aspects of diagnosis and management of this rare disease. The high costs of investigation panels and therapeutic foods remain an obstacle for recognizing and treating this disease in a resource-poor setting. Moreover, GA-II in the neonatal form with or without congenital anomalies is said to be associated with poor prognosis and high mortality. Hence, further research is required for better treatment modalities.
